# RNA Sequencing Analysis of Gene Expression by Electroacupuncture in Guinea Pig Gallstone Models

**DOI:** 10.1155/2022/3793946

**Published:** 2022-01-07

**Authors:** Mingyao Hao, Zhiqiang Dou, Luyao Xu, Zongchen Shao, Hongwei Sun, Zhaofeng Li

**Affiliations:** ^1^External Treatment Center of Traditional Chinese Medicine, Affiliated Hospital of Shandong University of Traditional Chinese Medicine, Jinan 250014, China; ^2^College of Acupuncture and Moxibustion, Shandong University of Traditional Chinese Medicine, Jinan 250355, China; ^3^Institute of Acupuncture and Moxibustion, Shandong University of Traditional Chinese Medicine, Jinan 250355, China

## Abstract

**Background:**

Clinical studies have shown that electroacupuncture (EA) promotes gallbladder motility and alleviates gallstone. However, the mechanism underlying the effects of EA on gallstone is poorly understood. In this study, the mRNA transcriptome analysis was used to study the possible therapeutic targets of EA.

**Methods:**

Hartley SPF guinea pigs were employed for the gallstone models. Illumina NovaSeq 6000 platform was used for the RNA sequencing of guinea pig gallbladders in the normal group (Normal), gallstone model group (Model), and EA-treated group (EA). Differently expressed genes (DEGs) were examined separately in Model vs. Normal and EA vs. Model. DEGs reversed by EA were selected by comparing the DEGs of Model vs. Normal and EA vs. Model. Biological functions were enriched by gene ontology (GO) analysis. The protein-protein interaction (PPI) network was analyzed.

**Results:**

After 2 weeks of EA, 257 DEGs in Model vs. Normal and 1704 DEGs in EA vs. Model were identified. 94 DEGs reversed by EA were identified among these DEGs, including 28 reversed upregulated DEGs and 66 reversed downregulated DEGs. By PPI network analysis, 10 hub genes were found by Cytohubba plugin of Cytoscape. Quantitative real-time PCR (qRT-PCR) verified the changes.

**Conclusion:**

We identified a few GOs and genes that might play key roles in the treatment of gallstone. This study may help understand the therapeutic mechanism of EA for gallstone.

## 1. Introduction

Gallstone disease affects 4% to 20% of the population [1, 2]. More than 80% of gallstones were cholesterol stones [3, 4]. Though approximately 3/4 of the patients are asymptomatic, the major burden generates when symptoms or complications occur [5]. Therefore, it has gained attention worldwide [2, 6]. Cholecystectomy is recommended to be offered to patients with symptomatic gallbladder stones [7]. However, patients hesitate or are unwilling to consider cholecystectomy because of the fear of potential risks and the concern for postoperative complications. The pursuit of physical integrity in some cultural backgrounds also limits its application. Although gallbladder-preserving cholelithotomy preserves gallbladder function and reduces surgical complications, the problem of stone recurrence is still an unsolvable problem.

The mechanisms of the pathogenesis of gallstone are multifaceted, including lithogenic genes, altered bile lipid composition, intestinal absorption of cholesterol, gut microbiota, defective gallbladder motility, dietary factors, and lifestyles [3, 5, 8]. Diet style has been considered one of the risk factors of gallstone disease. A high-lipid diet or fat consumption from meat or fried foods increases the likelihood of gallstones [8, 9]. While diet patterns, such as vegetarian diet, alternate Mediterranean diet, Alternate Healthy Eating Index diet, and Dietary Approaches to Stop Hypertension, may act as protective factors against the formation of gallstones [8, 10, 11]. Hence, a lithogenic diet is used as a common method to form gallstone animal models. Studies have found that some genes, such as mucin genes and lith genes, were associated with gallstone formation [12–15]. Recently, gut microbiome studies have shown that they have a close correlation with many disease conditions. Hence, great attention has been gained to the involvement of the gut microbiome in gallstone pathogenesis. The gut microbiome promotes the formation of gallstones by affecting gallbladder motility, affecting cholesterol metabolism and secretion, affecting bile acid metabolism, and eliciting chronic inflammation [16]. 16S rRNA gene sequencing analysis found that gallstone mice models displayed reduced microbiota richness. Lower Firmicutes level and decreased Firmicutes/Bacteroidetes ratio might be significant factors for the gallstone formation [17].

The hypomotility of the gallbladder is also believed to be one of the key causes of gallstones [18, 19]. Gallbladder hypomotility delays emptying, increases residual volume, and reduces its response to CCK, resulting in cholestasis, bile cholesterol supersaturation, the further aggravation of gallbladder hypomotility, and the promotion of the formation of gallstones [20]. Studies have shown that postprandial hypomotility of the gallbladder is an independent risk factor for gallstone recurrence after lithotripsy [18]. Therefore, improving gallbladder motility may be the key to the treatment of gallstones. The previous studies [21, 22] of our research team showed that the gallbladder relaxed on a large scale after accepting acupuncture. The clinical trials and animal studies suggest that acupuncture on Yanglingquan (GB34), Qimen (LR14), and Yidan (CO11) is an effective method for regulating gallbladder motility [23, 24]. Yet, the underlying mechanisms of acupuncture for gallstones remain largely unclear.

High-throughput RNA sequencing (RNA-seq) has been widely used to explore the mechanisms of a series of diseases. However, only a few studies have been carried out on gallstone gene expression. Therefore, the present study was carried out to explore the potential complicated molecular mechanisms of EA in gallstone by RNA-seq to identify key genes. We generated a gallstone guinea pig model and performed RNA-seq to analyze mRNA profiles in the gallbladder tissues of the guinea pigs from the Normal group, Model group, and EA group. DEGs were investigated in the Model vs. Normal and EA vs. Model separately. Enrichment analysis and PPI analysis were performed to explore the potential mechanism in gallstone. Hub genes were selected to verify RNA expression by qRT-PCR.

## 2. Materials and Methods

### 2.1. Animal Experiments

30 male SPF Hartley guinea pigs (250 g to 300 g, 4 to 5 weeks) were purchased from Qingdao Kangda Biotechnology Co., Ltd (Certification No. SCXK 20160002). The Ethical Committee for Research involving Animals of SDUTCM approved all procedures (20190125001). All animals were maintained in the Animal Experiment Center of the Affiliated Hospital of Shandong University of Traditional Chinese Medicine. After 1 week of acclimation and normal diet feeding, they were randomly divided into 3 groups (*n* = 10), namely (1) Normal group, (2) Model group, and (3) EA group. The Normal group was fed with a normal diet, while the Model group and EA group were fed with a lithogenic diet (purchased from Jiangsu Xietong Pharmaceutical Bioengineering Co., Ltd) for 6 weeks. After modeling, the gallbladder of all guinea pigs in the Model group and the EA group were observed under ultrasound examination (M5Vet, Mindray, Shenzhen, China) to verify the success of the gallstone model.

### 2.2. EA Treatment

**Figure 1:**
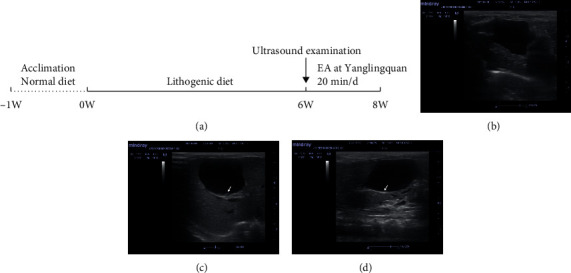
Representation of the flow chart (a) and the ultrasound examinations of guinea pig gallbladders from the Normal group (b), the Model group (c), and the EA group (d) separately after 6 weeks of lithogenic diet feeding. The arrow in (c) and (d) shows gallstones in gallbladder. EA: electroacupuncture.

EA treatment was provided for guinea pigs in the EA group after the gallstone model was established. Stainless steel acupuncture needles (Hwato, 0.25 mm × 13 mm, 200242) were inserted at a depth of 2 mm on both Yanglingquan (GB34) acupoints in the EA group. Then, the two acupuncture needles were connected with an EA stimulator (Hwato, SDZ-V) at an intensity range from 0.4 to 0.6 mA, a frequency of 20 Hz, and the continuous wave for 20 minutes once daily for 2 weeks (Figure 1(a)).

### 2.3. Sample Collection and Sequencing

All guinea pigs were sacrificed at the end of the EA treatment, and the gallbladders were collected. The samples were quickly put into liquid nitrogen for temporary storage and removed to a −80°C refrigerator for long-term storage. 3 gallbladders were randomly chosen from each group to do RNA-seq analysis. Total RNA was extracted by TRIzol (Invitrogen, United States). RNA samples were further purified with magnetic oligo (dT) beads after denaturation. Purified mRNA samples were reverse transcribed to first-strand cDNA, and a second cDNA was further synthesized. Fragmented DNA samples were blunt-ended and adenylated at the 3′ ends. Adaptors were ligated to construct a library. DNA was quantified by Qubit (Invitrogen). After cBot cluster generation, DNA samples were sequenced using Illumina NovaSeq 6000 (Illumina, San Diego, CA, United States) from Genergy Bio Technology (Shanghai, China).

### 2.4. RNA-Seq Analysis

Raw data were converted to Fastq format. Quality control (QC) was performed by FastQC (version 0.11.5) (https://www.bioinformatics.babraham.ac.uk/projects/fastqc/). STAR [25] (2.5.3a) (https://github.com/alexdobin/STAR) was used to map the clean reads to the reference genome (ftp://ftp.ensembl.org/pub/release99/fasta/cavia_porcellus/dna/Cavia_porcellus.Cavpor3.0.dna.toplevel.fa.gz). StringTie [26] (https://ccb.jhu.edu/software/stringtie/) was used to assess the expression levels of mRNAs by calculating FPKM. DESeq2 software [27] (v1.16.1) (https://bioconductor.org/packages/release/bioc/html/DESeq2.html) was used to calculate DEGs between different samples with *P* < 0.05 and |log2FC| ≥ 1 as the threshold. DEGs reversed by EA were identified. DEGs upregulated in Model vs. Normal but downregulated in EA vs. Model were defined as DEGs reversed downregulated (Reversed DOWN DEGs), and DEGs downregulated in Model vs. Normal but upregulated in EA vs. Model were defined as DEGs reversed upregulated (Reversed UP DEGs). Reversed UP DEGs and Reversed DOWN DEGs were defined as EA reversed DEGs.

### 2.5. Bioinformatics Analysis

GO enrichment was analyzed in EA reversed DEGs by the DAVID database [28,29] (https://david.ncifcrf.gov/home.jsp). The PPI network of EA-reversed DEGs was predicted and constructed using the STRING online database [30] (https://string-db.org). Then, Cytoscape software [31] (v3.8.1) (https://cytoscape.org/) was applied to visualize the network and distinguish the hub genes.

### 2.6. Quantitative Real-Time PCR

Total RNA was extracted from the samples of gallbladders with TRIzol (Invitrogen, United States) according to the manufacturer's instructions. Then RNA was converted to cDNA using *EasyScript*® One-Step gDNA Removal and cDNA Synthesis SuperMix (AE311-02, TransGen Biotech, Beijing, China). The level of transcripts was determined by qRT-PCR using the *PerfectStart*® Green qPCR SuperMix (AQ601-02, TransGen Biotech, Beijing, China) on an Applied Biosystems QuantStudio™ 5 Real-Time PCR Instrument (Thermo Fisher Scientific, United States). Primers were obtained from Sangong Biotech (Shanghai, China). The sequences of E2F1 are GCAGCAACTGGACCACCTAA (Forward primer) and AAGACATCGATGGGGCCTTG (Reverse primer). The sequences of GAPDH are GCTGATGCCCCTATGTTCGT (forward primer) and TGATGGCATGGACTGTGGTC (reverse primer).

### 2.7. Statistical Analysis

The data of qRT-PCR were presented as mean ± standard deviation (SD) and were analyzed with one-way ANOVA followed by Tukey's multiple comparisons tests in GraphPad Prism 8.4.2. The value of *P* < 0.05 was considered statistically significant.

## 3. Results

### 3.1. Model Identification of Gallstone

The gallstone guinea pig model was established by lithogenic diet. Abdominal ultrasound examination showed gallstones in the Model group and the EA group after 6 weeks of lithogenic diet (see Figures 1(b)–1(d)).

### 3.2. Quality Assessment

More than 30 million reads of each sample were obtained, and the percentages of Q20 and Q 30 were above 97% and 93%, respectively, in each sample. These results indicated that the quality of the sequencing was acceptable. More than 70% of clean reads were mapped onto the reference genome (see Table 1).

### 3.3. Identification of DEGs

**Figure 2:**
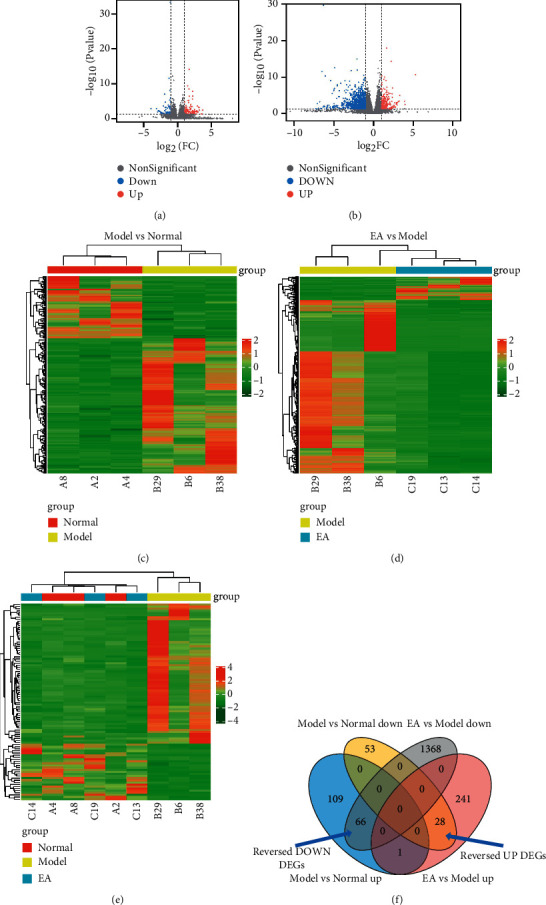
DEGs identified: volcano plots (a) and heatmap (c) of 257 DEGs in Model vs. Normal, with 176 upregulated DEGs and 81 downregulated DEGs. Volcano plots (b) and heatmap (d) of 1704 DEGs in EA vs. Model, with 270 upregulated DEGs and 1434 downregulated DEGs. A heatmap of 94 reversed DEGs (e). Venn diagram of EA reversed DEGs (f). Normal: the normal group, model: the model group, EA: the electroacupuncture group, DEGs: differently expressed genes, reversed DOWN DEGs: electroacupuncture-reversed downregulated differently expressed genes, and reversed UP DEGs: electroacupuncture-reversed upregulated differently expressed genes.

We used Illumina NovaSeq 6000 to analyze the gallbladder tissues of guinea pigs after 2-week electroacupuncture. The data were analyzed and DEGs were selected according to the threshold of *P* < 0.05 and |log2FC| ≥ 1. 257 DEGs were identified in Model vs. Normal, with 176 upregulated genes and 81 downregulated genes (see Figure 2(a), Figure 2(c), and Table S1). 1704 DEGs were found in EA vs. Model, with 270 upregulated DEGs and 1434 downregulated DEGs (see Figures 2(b), 2(d), and Table S2). Among these DEGs, 94 EA-reversed DEGs were identified, including 28 reversed UP DEGs and 66 reversed DOWN DEGs (see Figure 2(e), Figure 2(f), and Table S3).

### 3.4. Go Enrichment Analysis of 94 EA Reversed DEGs

**Figure 3:**
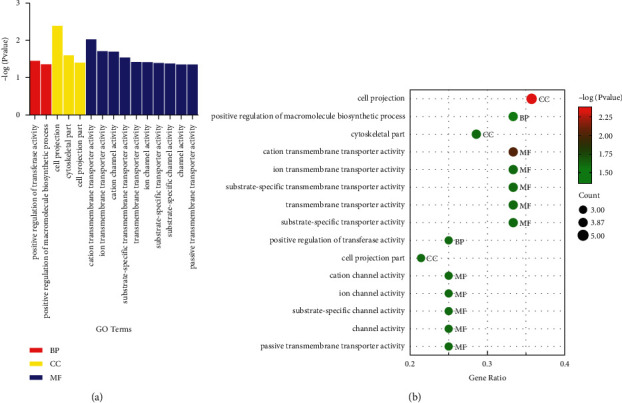
GO analysis of 94 EA reversed DEGs. Bar plot (a) of EA-reversed DEGs showed an enrichment score (-log(*P* value)) of the significant enrichment terms involving all 2 BPs, 3 CCs, and 10 MFs. Each bubble in the bubble plot (b) represents different -log(*P* value). GO: gene ontology; DEGs: differently expressed genes; BP: biological process; CC: cellular component; MF: molecular function.

GO enrichment analysis (including BP, biological process; CC, cellular component; MF, molecular function) was performed to predict the underlying biological functions of 94 EA reversed DEGs. The DEGs were enriched to 15 GO terms, including 2 BPs, 3 CCs, and 10 MFs (*P* < 0.05). The 2 BPs were positive regulation of transferase activity, and positive regulation of macromolecule biosynthetic process. The 3 CCs were cell projection, cytoskeletal part, and cell projection part. The 10 MFs were cation transmembrane transporter activity, ion transmembrane transporter activity, cation channel activity, substrate-specific transmembrane transporter activity, transmembrane transporter activity, ion channel activity, substrate-specific transporter activity, substrate-specific channel activity, channel activity, and passive transmembrane transporter activity as shown in Table 2 and Figure 3.

### 3.5. Construction of the PPI Network and Identification of Hub Genes

**Figure 4:**
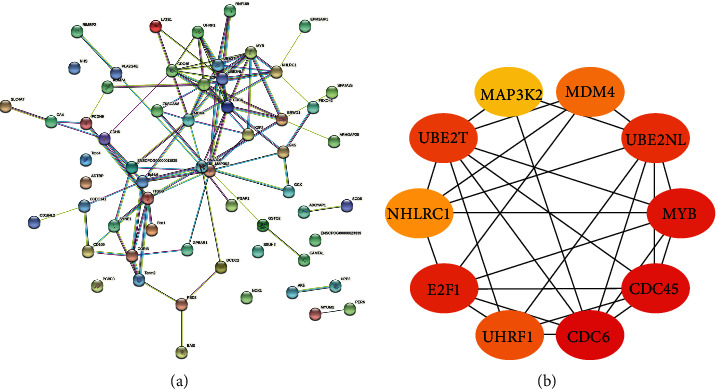
PPI network of 94 EA reversed DEGs (a) with a score >0.150, and the top 10 hub genes of EA-reversed DEGs by the cytohubba plugin (b). Each node stands for a gene or a protein, and the edges represent the interactions between the nodes. PPI: protein-protein interaction; DEGs: differently expressed genes.

94 EA-reversed DEGs were used to construct the PPI network based on the STRING database to identify the hub genes that may play key roles in the EA effect of gallstones. 59 nodes and 98 edges were established in the PPI network with score >0.150 (Figure 4(a)), and 46 nodes and 95 edges were analyzed. Cytohubba plugin was used to find the hub genes. The top 10 hub genes were CDC6, CDC45, MYB, E2F1, UBE2NL, UBE2T, UHRF1, MDM4, NHLRC1, and MAP3K2 (see Figure 4(b)).

### 3.6. Gene Validation

**Figure 5:**
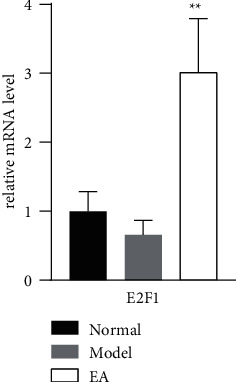
E2F1 mRNA expression using qRT-PCR. Statistical analysis was performed with one-way analysis of variance (ANOVA) and Tukey's multiple comparisons tests by GraphPad Prism 8.4.2. Compared with the Model group, ^*∗∗*^*P* < 0.01.

We randomly selected the E2F1 gene and performed qRT-PCR analysis to validate the expression of genes to confirm the key genes identified above. E2F1 was downregulated in Model vs. Normal and upregulated in EA vs. Model (*P* < 0.05), which validated the same trend in sequencing (see Figure 5).

## 4. Discussion

Lithogenic diet has been used widely in inducing animal gallstone models [32–34]. After successfully inducing the guinea pig gallstone models, we used high throughput RNA-seq to analyze the mRNA expression in guinea pig gallbladders of the Normal, Model, and EA groups. 257 DEGs and 1704 DEGs were separately identified in Model vs. Normal and in EA vs. Model. Among these DEGs, there are 94 EA-reversed genes, including 28 reversed UP DEGs and 66 reversed DOWN DEGs. We also predicted the potential functions of these EA-reversed DEGs using GO and PPI network analysis. Go enrichment analysis revealed that EA mainly regulated several GO-MF terms about the transmembrane transporter activities and channel activities.

Transporters are expressed in many tissues within the body and play important roles in human physiology, pharmacology, pathology, and toxicology [35]. EA involves multiple pathways and produces multitarget effects. In gallstone formation, cholesterol and its membrane transports were considered major pathogenic factors [5, 36]. Therefore, EA may involve in the transmembrane transporter activities of cholesterol and the regulation of glucose and lipid to reduce the gallstones. In our results, hub genes E2F1, UBE2T, UBE2NL, and UHRF1 involve in several important bioprocesses, including cell cycle progression, membrane transporting, regulation of cholesterol, glucose, lipid, etc.

E2F1 is a transcription factor that involves in cell cycle progression, DNA-damage response, and apoptosis [37, 38]. It also participates in the regulation of metabolism. The loss of E2F1 leads to abnormal cholesterol accumulation in the liver and the development of fibrosis in response to a high-cholesterol diet [39]. E2F1 regulates cholesterol uptake. By enhancing the expression of PCSK9, a negative regulator of cholesterol uptake, E2F1 increased cholesterol uptake [38, 39]. Cholesterol uptake is considered one of the factors for gallstones [5]. In our study, E2F1 expression was reduced in the Model group. However, it could be reversed to increase in the EA group. The result suggested that E2F1 might be correlated with the mechanism of gallstone, and it might be a possible therapeutic target of EA.

Research has shown that UBE2T, as a member of the E2 family in the ubiquitin-proteasome pathway, was key in protein ubiquitination. Ubiquitination controls diverse biological processes, including inflammation, immune response, cell differentiation, cell proliferation, etc. [40–45]. Dysregulated ubiquitin system may lead to a variety of diseases, such as various types of cancer, neurodegeneration, and metabolic disorders [46, 47]. In ubiquitination, E1, E2, and E3 enzymes sequentially activate, conjugate, and ligate ubiquitin to substrate proteins [48]. The E2s accept ubiquitin from the E1 complex and catalyze its covalent attachment to other proteins, involving in the change of protein stability, cellular localization, and biological activity. Hence, the E2 family is crucial in a wide range of biological processes, such as controlling the cell cycle, transducing signals, and inducing tumors, and it may provide an understanding of the pathogenesis of diseases [49]. As a member of the E2 family, UBE2T can induce cell cycle arrest at the G2/M phase and increase cell apoptosis. It has been reported to have a close relationship with the gallbladder. By analyzing microarray data, UBE2T was considered a hub gene in patients with gallbladder cancer, and it might serve as a biomarker for gallbladder cancer [50]. Through the PI3K/Akt signaling pathway and the Akt/GSK3*β*/*β*-catenin pathway, UBE2T is involved in the cell proliferation, migration, and invasion of cancer cells [51–53]. UBE2NL, also a ubiquitin-conjugating enzyme E2 family member, is related to the ubiquitination process [54]. It binds to ubiquitin-conjugating enzyme E2V2 [55–57] and interacts with E3 ubiquitin ligase in the polyubiquitination reaction and cell cycle progression [58]. UBE2NL has been found to be a novel type 2 diabetes relevant gene [59] and a novel candidate gene in familial gastroschisis [60]. It also expresses in the brain and participates in parkin-mediated mitophagy as a genetic risk factor for sporadic Alzheimer's disease [54].

UHRF1 is an E3 ubiquitin ligase that plays a key role in DNA methylation, DNA-damage repair, and cell proliferation [61]. UHRF1 is a key regulator of DNA double-strand break repair that directly participates in the interplay between VRCA1 and 53BP1 [62]. UHRF1 is important in epigenetics. During DNA replication, UHRF1 inherits DNA methylation. By binding either H3K9me2/3 or hemimethylated CpG, UHRF1 is required in the recruitment of DNMT1 to DNA methylation sites. It also maintains DNA methylation at genomic sites containing methylated H3K9 [63–65]. As an epigenetic regulator, UHRF1 is significant in vascular smooth muscle cell plasticity. By promoting proliferation and differentiation, UHRF1 regulates the VSMC phenotype, and it may hold therapeutic potential in vascular pathologies [66]. Silencing UHRF1 inhibits cell proliferation and promotes cell apoptosis in retinoblastoma through the PI3K/Akt signaling pathway [67]. UHRF1 is highly expressed in proliferating and cancer cells, and it has been identified as a novel AMPK gate-keeper in cellular metabolism by interacting with AMPK and suppressing its activity. UHRF1 is physiologically significant in the regulation of glucose and lipid [68], and thereby, it is involved in the regulation of liver metabolism. The liver overexpression of the UHRF1 mice model showed increasing blood glucose level, reducing glucose tolerance, reducing insulin sensitivity, and accumulating lipid droplets in the liver tissues. Gluconeogenesis, glycogen synthesis, and triglyceride synthesis-related gene upregulation is caused by overexpression [69].

The involvement of hub genes in the cholesterol, glucose and lipid metabolism gives a hint that EA may treat gallstone by regulating the metabolism. And it needs further design relevant experiments to verify these hypotheses. And There are limitations in this study. Due to lack of KEGG pathway of guinea pig in DAVID database, the KEGG enrichment analysis has not been carried out. And it also lacks the control group of sham acupuncture group. In the clinical practice, not only Yanglingquan (GB34) selected for the treatment of gallstone, but for the reason of standardization, we only use Yanglingquan (GB34) as the acupoint for EA. We have identified hub genes involved in the EA treatment of gallstone and may help to understand the therapeutic mechanism of EA. However, Further experiments need to be carried to study the expressions and interactions.

## 5. Conclusions

We analyzed the mRNA expression in gallbladders of guinea pigs in the Normal group, Model group, and EA group by high throughput RNA-seq. A number of key genes and GO terms were involved. These findings provide a clue to understand the possible therapeutic mechanism of EA on gallstone.

## Figures and Tables

**Table 1:** Quality assessment of the RNA-seq.

Sample	Raw reads	Clean reads	Clean reads (%)	Q20 (%)	Q30 (%)	Total mapped	Mapped ratio (%)
Normal 2	34014866	33102392	97.32	98.00	94.20	30576715	92.40
Normal 4	36119610	35112328	97.21	97.95	94.15	32305432	92.00
Normal 8	36385004	35490310	97.54	97.80	93.80	30009483	84.60
Model 6	32884164	31919142	97.07	98.00	94.15	29430412	92.20
Model 29	35567182	34572958	97.20	97.90	93.95	32029940	92.60
Model 38	32026472	30888052	96.45	97.85	93.90	28575186	92.50
EA 13	33984346	33147418	97.54	97.80	93.65	26207366	79.10
EA 14	36185162	34789908	96.14	97.95	94.15	24475529	70.40
EA 19	43398886	41221630	94.98	97.80	93.85	32803154	79.60

Q20: quality score of 20; Q30: quality score of 30; Normal: the normal group; Model: the model group; EA: the electroacupuncture group.

**Table 2:** GO terms of EA-reversed DEGs.

GO ID	GO term	GO category	*P* value
GO:0051347	Positive regulation of transferase activity	BP	0.03526
GO:0010557	Positive regulation of macromolecule biosynthetic process	BP	0.04364
GO:0042995	Cell projection	CC	0.00410
GO:0044430	Cytoskeletal part	CC	0.02518
GO:0044463	Cell projection part	CC	0.03979
GO:0008324	Cation transmembrane transporter activity	MF	0.00937
GO:0015075	Ion transmembrane transporter activity	MF	0.01942
GO:0005261	Cation channel activity	MF	0.01998
GO:0022891	Substrate-specific transmembrane transporter activity	MF	0.02899
GO:0022857	Transmembrane transporter activity	MF	0.03767
GO:0005216	Ion channel activity	MF	0.03837
GO:0022892	Substrate-specific transporter activity	MF	0.04042
GO:0022838	Substrate-specific channel activity	MF	0.04162
GO:0015267	Channel activity	MF	0.04441
GO:0022803	Passive transmembrane transporter activity	MF	0.04441

## Data Availability

The raw data can be available from the corresponding author on reasonable request.

## Supplementary Materials

Supplementary Materials Supplementary tables are provided. See Table S1 for the list of 257 DEGs in Model vs. Normal. See Table S2 for the list of 1704 DEGs in EA vs. Model. See Table S3 for 94 EA reversed DEGs.
